# N-terminal lysine as a critical determinant of odilorhabdin susceptibility to inactivation by the self-resistance acetyltransferase OatA

**DOI:** 10.1128/aac.00743-26

**Published:** 2026-09-16

**Authors:** Emilie Racine, Anne Lanois-Nouri, Jessica Houard, Lucile Pantel, Alain Givaudan, Maxime Gualtieri

**Affiliations:** 1 Nosopharm 381197 , Nîmes, France; 2 DGIMI, Univ Montpellier, INRAE, Montpellier, France; 3 UPR CHROME, Nîmes University, Nîmes, France; Entasis, Big Bay, Michigan, USA

**Keywords:** odilorhabdins, OatA acetyltransferase, enzymatic inactivation, self-resistance, structure-activity relationship, *Xenorhabdus nematophila*

## Abstract

Odilorhabdins (ODLs) are a novel class of peptide antibiotics that inhibit bacterial translation by binding to the 30S ribosomal subunit. *Xenorhabdus nematophila*, an ODL-producing bacterium, encodes a highly specialized self-resistance acetyltransferase, OatA, that inactivates ODLs via primary amine modification. In this study, we investigated the structural determinants governing susceptibility of the natural compound NOSO-95C to OatA-mediated inactivation. Using an alanine-scanning library, we identified *N*-terminal lysine (Lys_1_) as the key structural determinant of acetyltransferase susceptibility. Expression of *oatA* in *E. coli* increased the MIC of NOSO-95C by 64-fold due to rapid OatA-mediated acetylation. In contrast, analog **1** (Lys1→Ala) was no longer discriminated between Oat-A expressing and non-expressing strains, and remained 95.4% unacetylated after 4 h of incubation. Functional assays confirmed that analog **1** retains potent translation inhibition and antibacterial activity despite enzyme exposure. Furthermore, OatA failed to inactivate other amine-containing clinical antibiotics such as aminoglycosides, penicillin, or polymyxins. These results indicate that the side chain of *N*-terminal lysine is essential for efficient OatA-mediated acetylation of ODLs. Moreover, this single residue can be modified to enable ODLs to evade enzymatic inactivation while preserving their primary mechanism of action. This work provides a clear structure-activity framework for the rational design of next-generation ODLs with enhanced stability against OatA-mediated enzymatic modification.

## INTRODUCTION

Natural products from environmental bacteria constitute a rich and chemically diverse reservoir of bioactive compounds and are historically an important source of first-in-class antibiotics with novel mechanisms of action (1). However, the evolutionary success of antibiotic-producing microorganisms relies on their ability to protect themselves from the toxic effects of their own metabolites (2). As a consequence, biosynthetic gene clusters (BGCs) of antibiotic producers frequently encode dedicated self-resistance mechanisms, which may involve target modification, active efflux, or enzymatic inactivation of the antibiotic molecule itself (3–5). These self-resistance determinants are not necessarily indicative of a broader clinical resistance risk (6), but understanding their substrate requirements is essential for the rational design of next-generation analogs.

**Fig 1:**
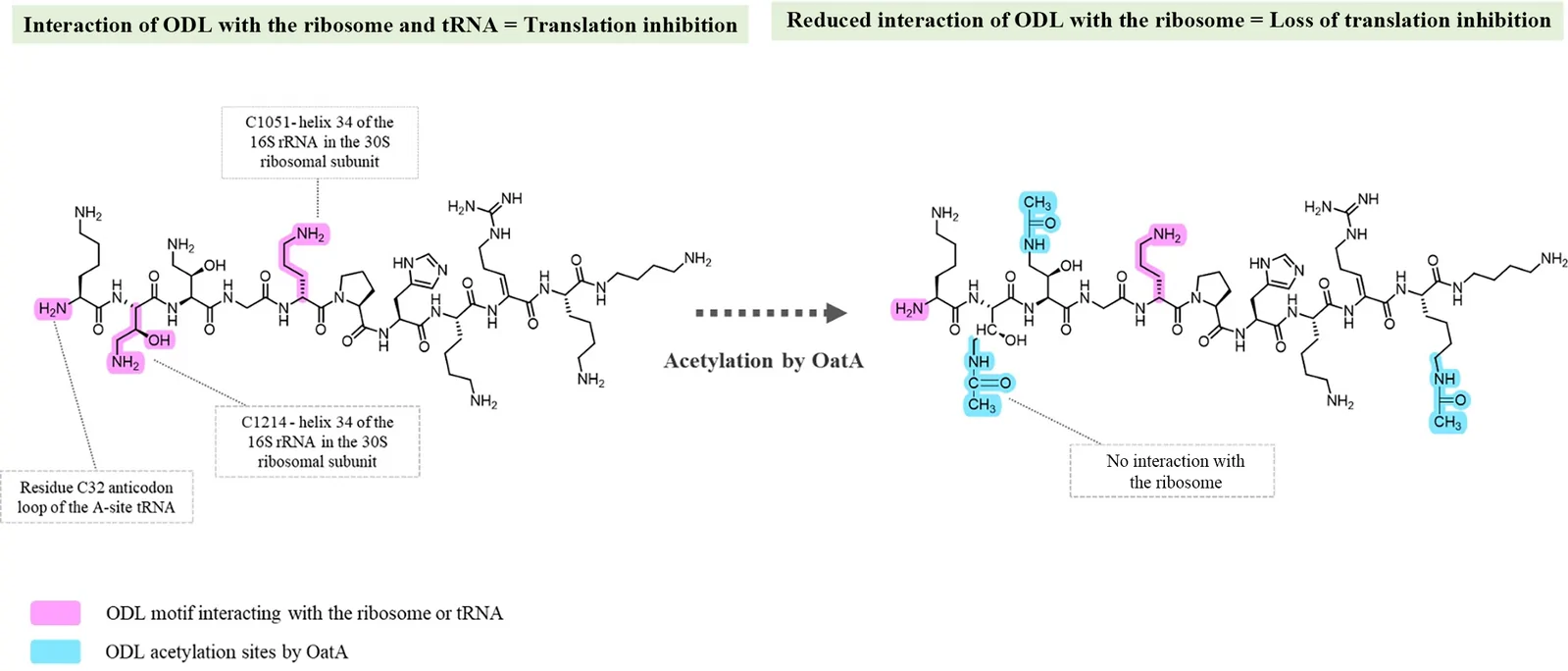
Mechanism of translation inhibition by odilorhabdins (ODLs) and impact of OatA-mediated acetylation. The scheme illustrates the binding motifs of ODLs (magenta) interacting with the 16S rRNA of the 30S ribosomal subunit and the A-site tRNA anticodon loop. To the right, acetylation by the OatA enzyme at specific primary amine sites (cyan) disrupts these ribosomal interactions, leading to a loss of protein synthesis inhibition (17).

*Xenorhabdus nematophila*, an entomopathogenic bacterium involved in a mutualistic relationship with *Steinernema carpocapsae* nematodes (7), produces a wide range of antimicrobial secondary metabolites, including odilorhabdins (ODLs) (8–10). ODLs are linear polycationic decapeptides assembled by nonribosomal peptide synthetases (NRPS), which are C-terminally modified with a α, δ-diamino butane polyamine moiety. They bind a unique site in the 30S ribosomal decoding center, contacting 16S rRNA and the A-site tRNA anticodon loop (Fig. 1), to induce miscoding and inhibit translocation (11). Medicinal chemistry optimization led to the development of NOSO-502, the first ODL candidate to complete regulatory preclinical development for the treatment of multidrug-resistant Enterobacteriaceae infections (12–14). Resistance to ODLs mechanistically involves either alterations of the ribosomal target (H56Y substitution on the S10 ribosomal protein) or reduced intracellular accumulation through efflux (11, 15, 16). *X. nematophila* itself relies on a producer-restricted self-resistance mechanism: the GNAT-family acetyltransferase OatA, encoded by *odlC* within the ODL BGC, inactivates ODLs by acetylating primary amines (17, 18). OatA has been shown to target Dab(βOH)2, Dab(βOH)3, and Lys10 (Fig. 1) (17), but the structural determinants governing substrate recognition remain unknown.

In this study, we used alanine scanning and N-terminal modifications of the natural ODL, NOSO-95C, to identify the moieties governing OatA-mediated acetylation, with the goal of defining modifications that allow ODL analogs to evade this self-resistance enzyme while retaining ribosomal target engagement. We selected an analog lacking the Lys1 side chain amine (analog 1) as a lead, showing no sensitivity to OatA activity, as demonstrated by comparable antibacterial activity against bacterial strains either expressing or lacking this enzyme. We then performed *in vitro* acetylation and liquid chromatography-tandem mass spectrometry (LC-MS/MS) analyses to confirm the non-impact of OatA against this analog. We then assessed the risk of cross-reactivity by testing OatA against a panel of clinically relevant amine-containing antibiotics. These findings provide a structural basis for designing next-generation ODLs that escape this inactivation mechanism.

## RESULTS

### Alanine scanning of NOSO-95C and identification of determinants of OatA susceptibility

The contribution of individual residues of NOSO-95C to its intrinsic antibacterial activity has been extensively characterized previously (12). In the present study, we therefore focused exclusively on the role of each position in susceptibility to OatA-mediated inactivation. To this end, an alanine-scanning library of NOSO-95C was used. Each amino acid was individually substituted with alanine, yielding a total of ten single-substitution analogs, complemented by a C-terminally truncated analog (analog **11**) (Table 1). All compounds were synthesized by solid-phase peptide synthesis following previously described methods (12). Antibacterial activity was assessed by determination of minimum inhibitory concentrations (MICs) against *Escherichia coli* XL1 carrying the empty vector pUC19 and against an isogenic *E. coli* XL1 strain harboring the *X. nematophila oatA* gene under the control of an inducible P*_lac_* promoter (P*_lac_-oatA*-Xn). This heterologous, IPTG-inducible expression system reproduces the enzyme activity level previously validated as sufficient to generate a reproducible resistance phenotype (17), allowing side-by-side comparison of the 10 analogs under identical induction conditions. NOSO-95C displayed a 64-fold OatA-dependent decrease in susceptibility, with a MIC of 4 µg/mL against *E. coli* XL1 (pUC19) and 256 µg/mL against *E. coli* XL1 (P*_lac_-oatA*-Xn) (Table 1). This resistance profile served as the reference for evaluating the impact of individual substitutions.

**TABLE 1:** Alanine-scanning library of NOSO-95C: impact on translation inhibition and susceptibility to OatA-mediated inactivation^*a*^

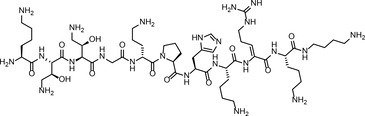 NOSO-95C: Lys_1_-Dab(βOH)_2_-Dab(βOH)_3_-Gly_4_-Orn_5_-Pro_6_-His_7_-Lys_8_-dhArg_9_-Lys_10_-1,4 Dbt_11_									
Analog ID and chemical structure		IVTT	MIC (µg/mL) against *E. coli* XL1		Analog ID and chemical structure		IVTT	MIC (µg/mL) against *E. coli* XL1	
		IC_50_ (µM)	− OatA	+ OatA			IC50 (µM)	− OatA	+ OatA
NOSO-95C	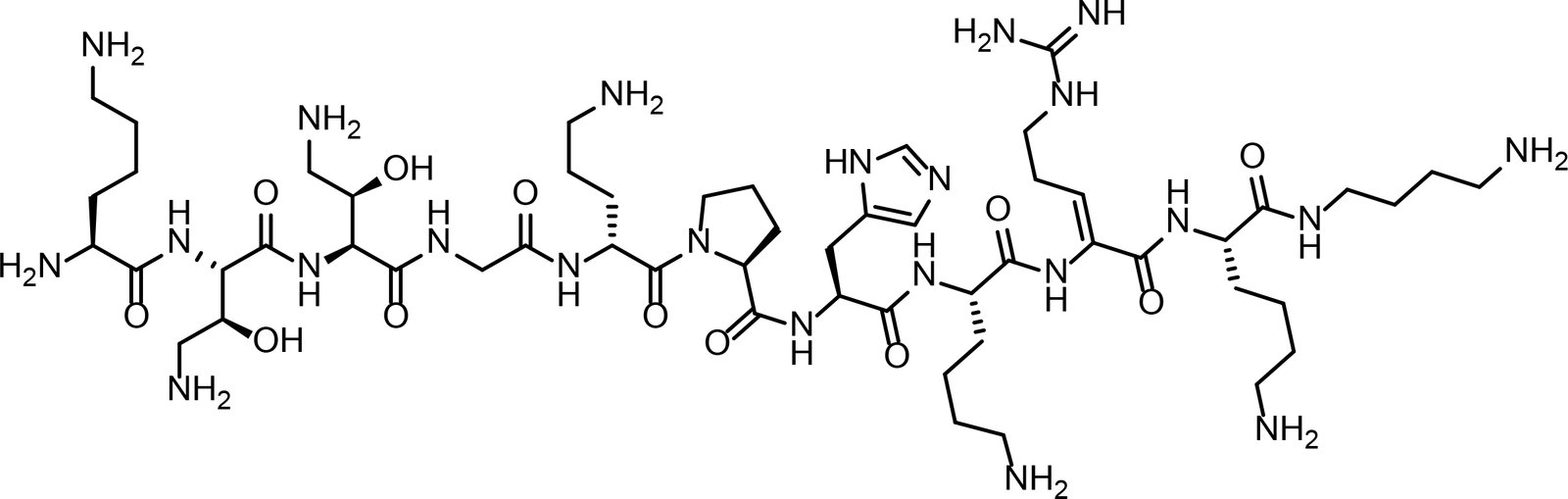	0.46	4	256 (× 64)	6	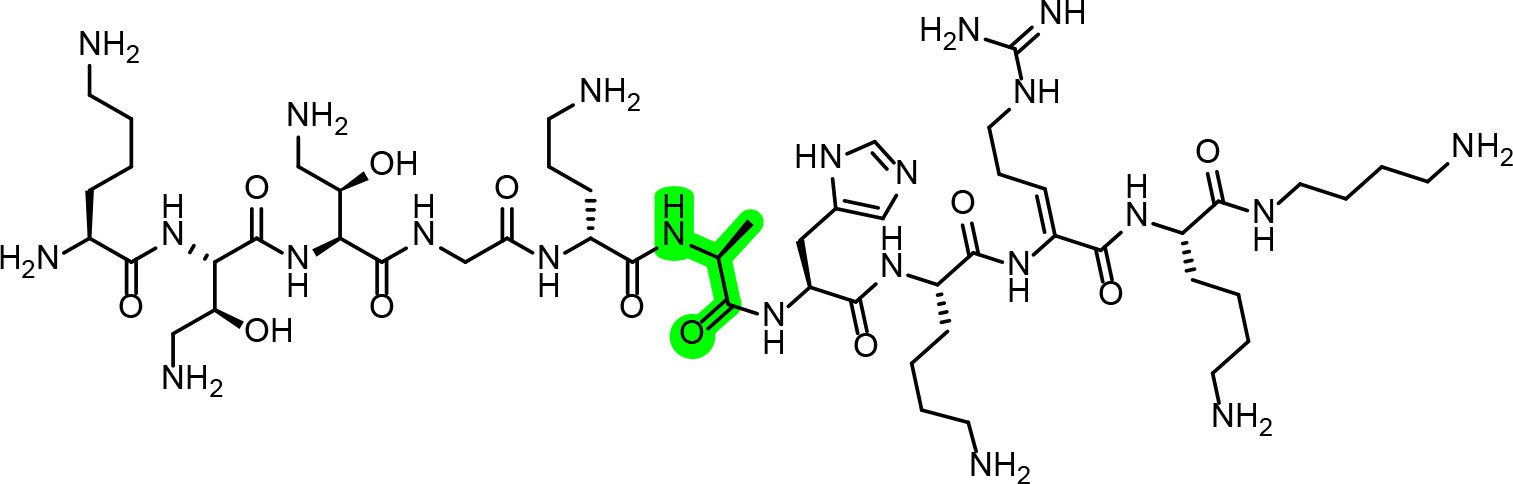	3.41	32	512 (× 16**)**
1	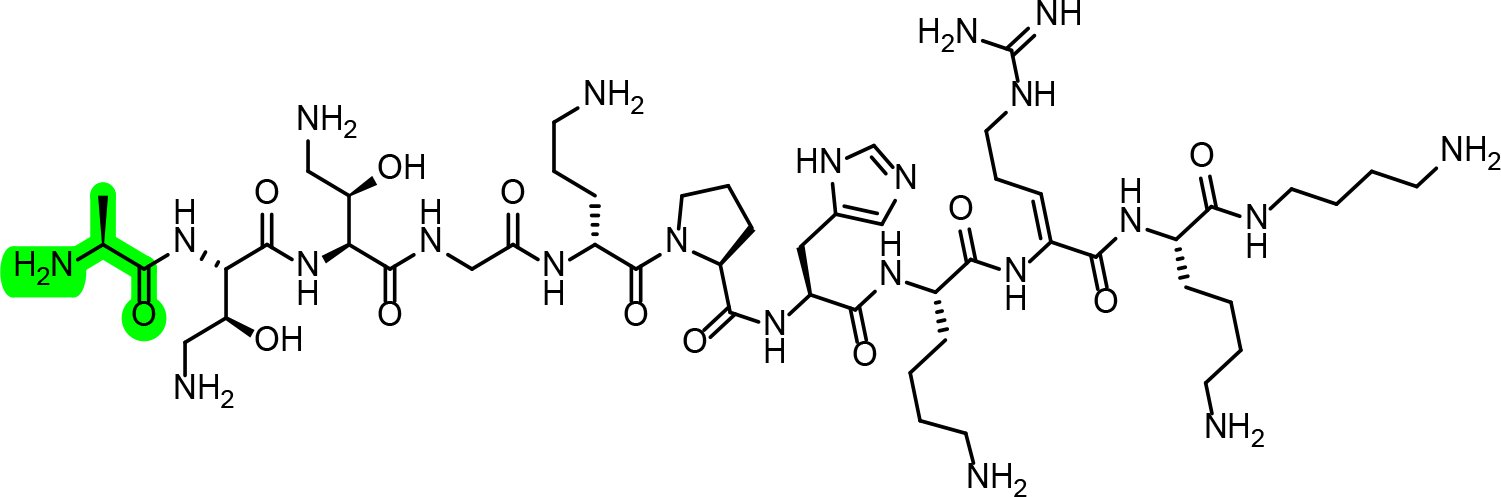	0.80	8	8 (× 1)	7	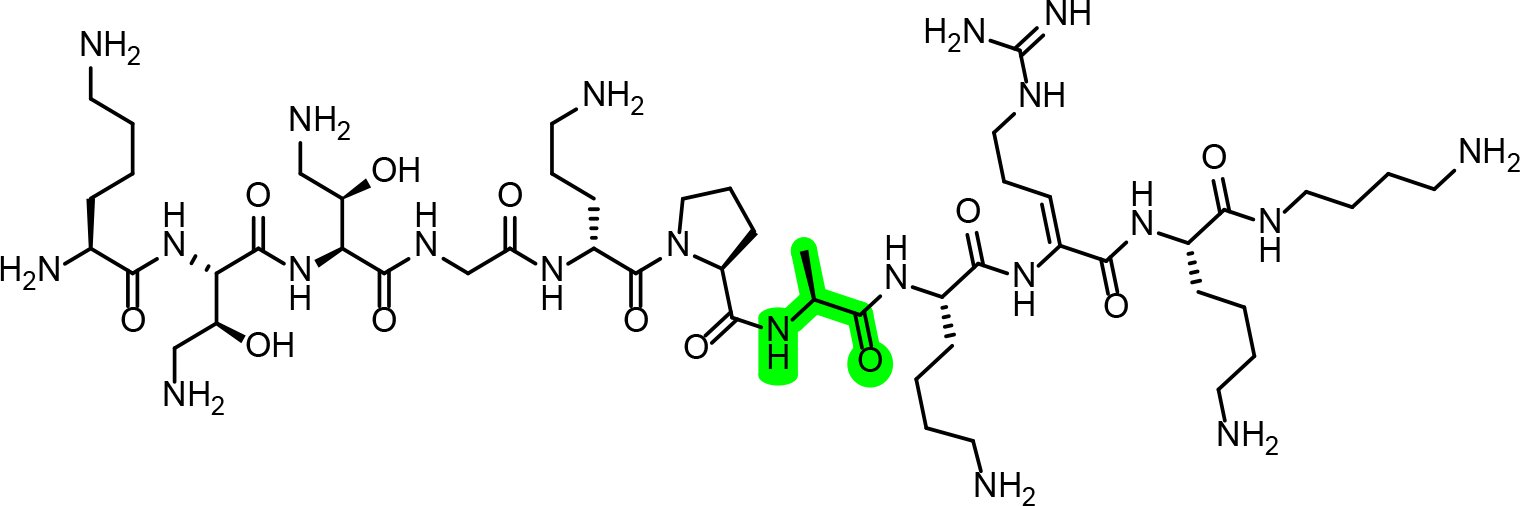	0.66	16	512 (× 32)
2	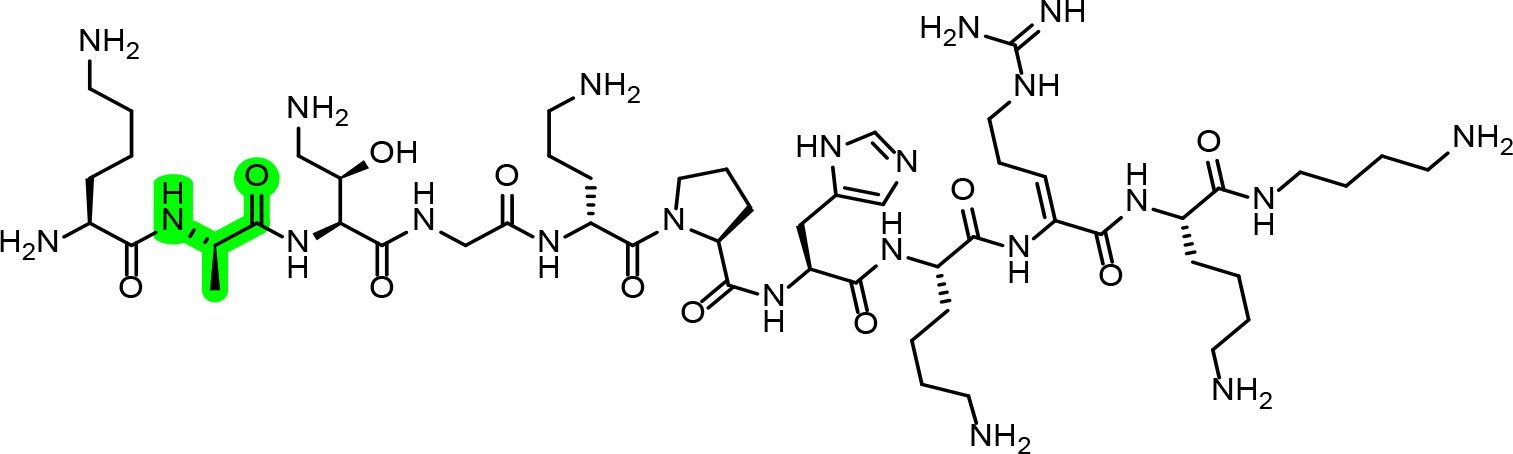	75.1	>2,048	>2,048 (nd)	8	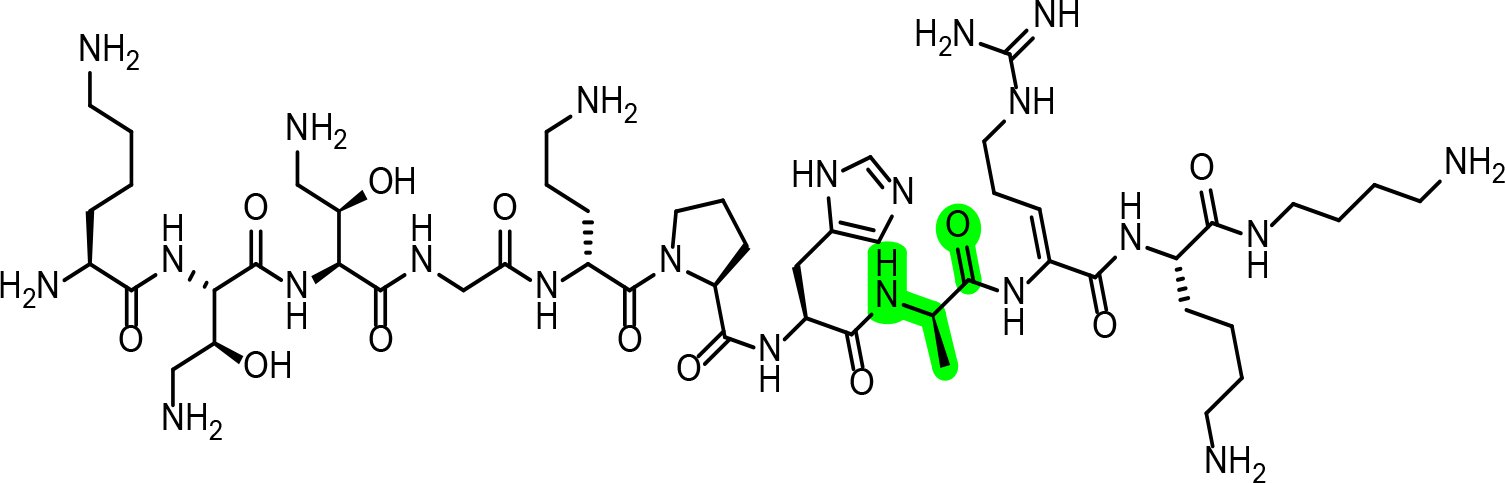	1.42	8	1,024 (× 128)
3	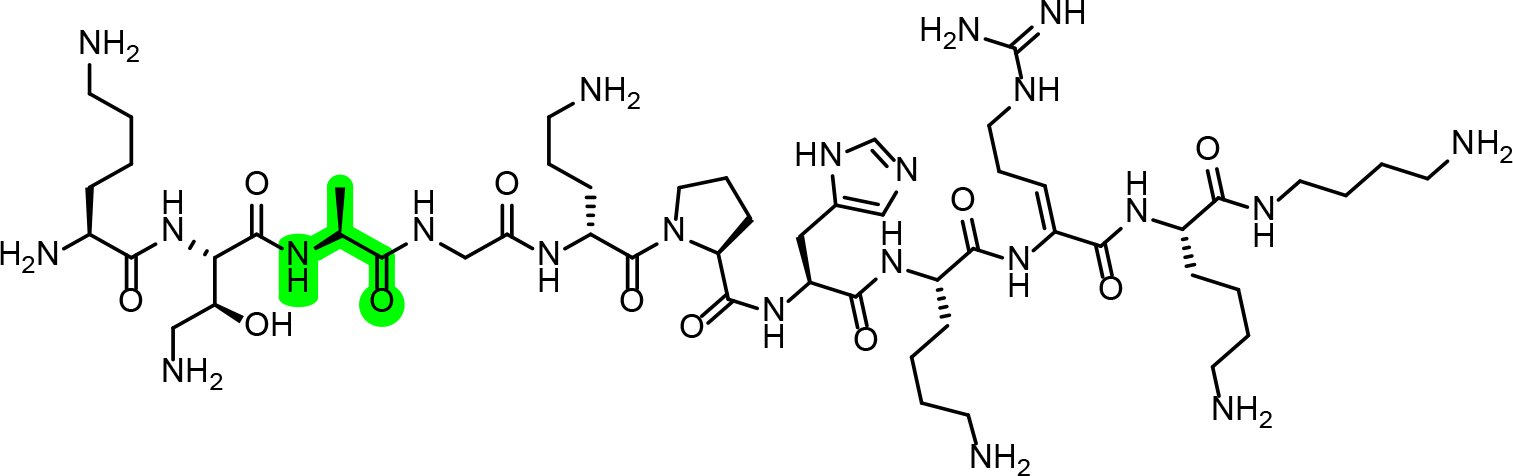	0.52	2	128 (× 64)	9	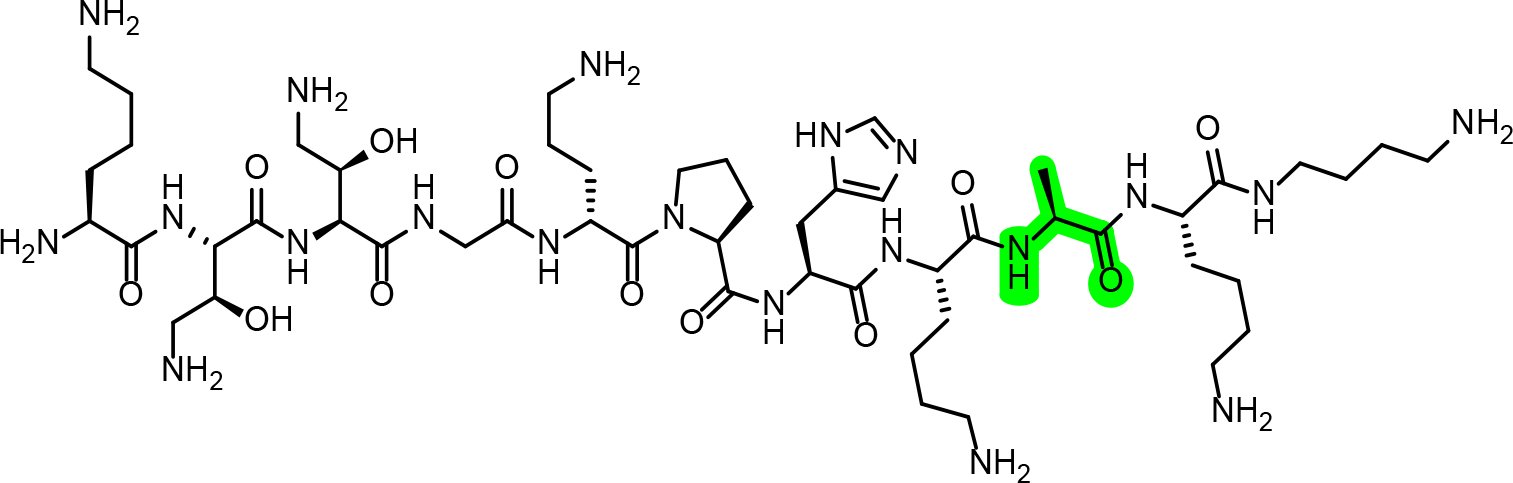	2.40	32	>2,048 (> ×64)
4	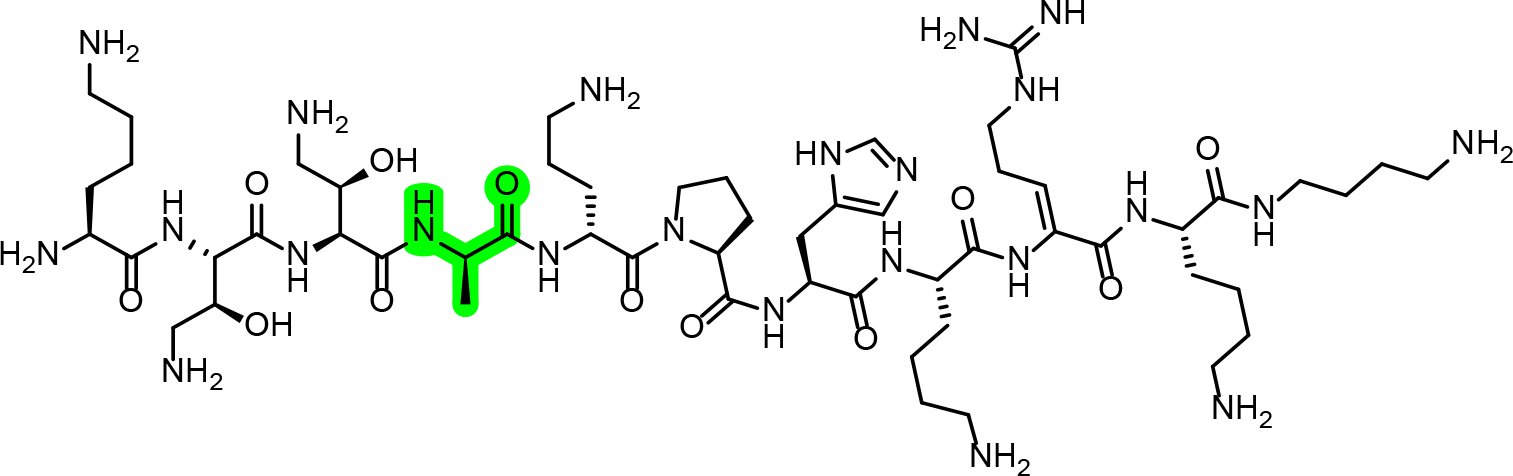	2.43	64	>2,048 (**>** ×32)	10	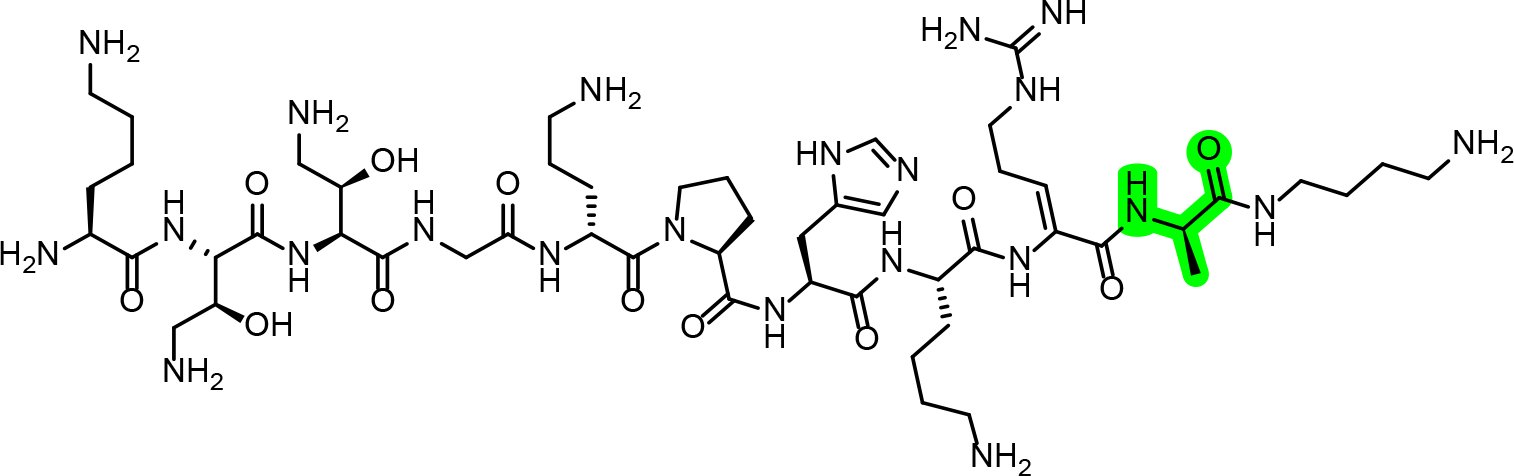	0.50	4	2,048 (× 512)
5	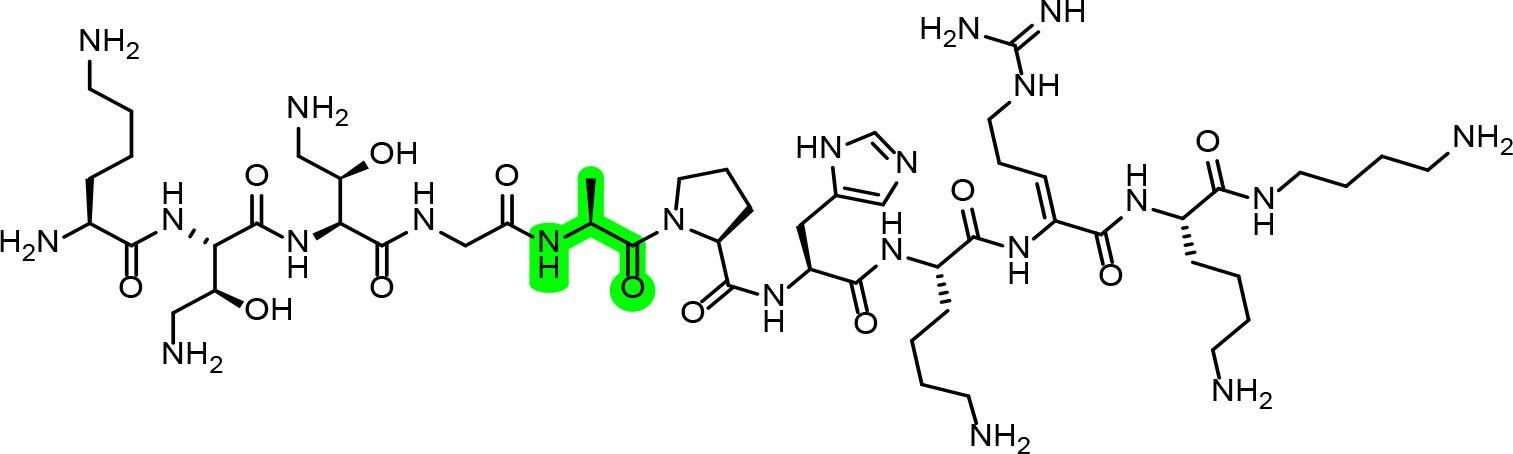	>142	1024	>2048 (**>** × 2)	11	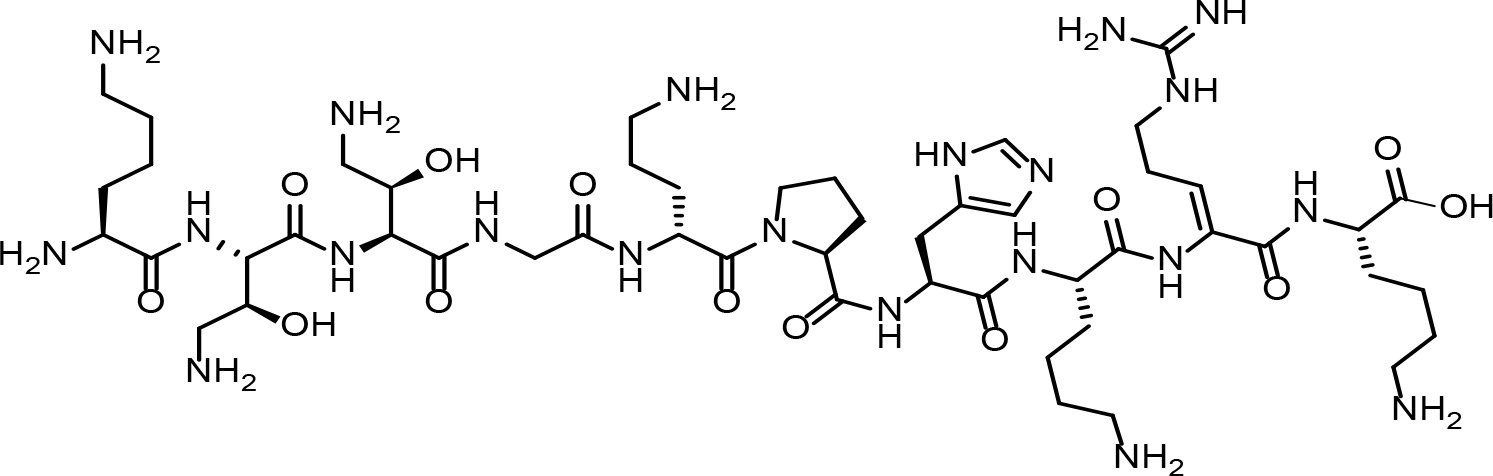	0.32	4	1024 (× 256)

^*a*^ Data include IC_50_ values determined by an *E. coli in vitro* transcription-translation (IVTT) assays and minimum inhibitory concentrations (MICs) against *E. coli* XL1 (pUC19) (– OatA) and against *E. coli* XL1 (P*_lac_-oatA*-Xn) (+ OatA). Values in red parentheses represent the MIC ratio (fold-increase) between OatA-expressing and non-expressing *E. coli* XL1 strains. nd, not determined.

Substitution of Lys1 with alanine (analog **1**) completely abolished OatA-mediated shift, yielding identical MICs of 8 µg/mL against both strains. Replacement of Dab(βOH)3 with alanine (analog **3**) preserved a 64-fold shift comparable to the parent compound, although with improved intrinsic activity (MIC = 2 µg/mL against the control strain). Substitution of Gly4 (analog 4) also preserved a substantial OatA-dependent shift (MIC of 64 µg/mL against the control strain versus >2,048 µg/mL against the OatA-expressing strain, corresponding to a >32-fold shift), indicating that this position is not required for OatA recognition. Substitution of Pro6 and His7 (analogs **6** and **7**) resulted in reduced but still significant OatA-dependent shifts of 16- and 32-fold, respectively. Analogs **8**, **9**, and **10**, bearing alanine substitutions at Lys8, Dha9, and Lys10, displayed shifts of 128-fold, >64-fold, and 512-fold, respectively. To further probe whether the C-terminal region of NOSO-95C, beyond single-residue substitution, contributes to enzyme recognition, we additionally generated analog 11, in which the C-terminal Dbt11 residue was removed rather than substituted with alanine. This truncation similarly retained a high level of OatA sensitivity, with a 256-fold shift (4 vs 1024 µg/mL), indicating that the C-terminal region is dispensable for OatA-mediated inactivation regardless of whether it is mutated or removed. As previously described (12), substitutions at positions 2 and 5 severely impaired intrinsic antibacterial activity (MIC >2,048 and 1,024 µg/mL, respectively, against the control strain), precluding meaningful assessment of their contribution to OatA-mediated inactivation (Table 1). In parallel, inhibition of bacterial translation was quantified for each compound by determining IC₅₀ values using an *in vitro* coupled transcription–translation assay based on *E. coli* cell extracts. This assay was used to confirm that the alanine analogs retained the ability to inhibit protein synthesis. IC₅₀ values of compounds with MIC <32 µg/mL against *E. coli* remained in a relatively narrow range (0.32–1.42 µM) (Table 1), confirming that the alanine substitutions tested did not broadly compromise ribosome engagement, in contrast to their heterogeneous effects on OatA susceptibility.

### Impact of N-terminal side chain length and amine functionality on susceptibility to OatA-mediated inactivation

To further investigate the role of the *N*-terminal residue, a series of analogs was generated by modifying Lys_1_. The first series aimed at decreasing the length of the lateral chain. Replacement of Lys_1_ with 2,4-diaminobutyric acid (analog **12**) or with ornithine (analog **13**) led to a slight decrease in OatA-mediated inactivation, as reflected by 4- and 8-fold MIC shifts, respectively, between an *E. coli* strain expressing or not expressing *oatA* (Table 2).

**TABLE 2:** Influence of *N*-terminal Lys1 modification on OatA-mediated inactivation^*a*^

Analog ID & chemical structure		IVTT	MIC (µg/mL) against *E. coli* XL1	
		IC_50_ (µM)	− OatA	+ OatA
12	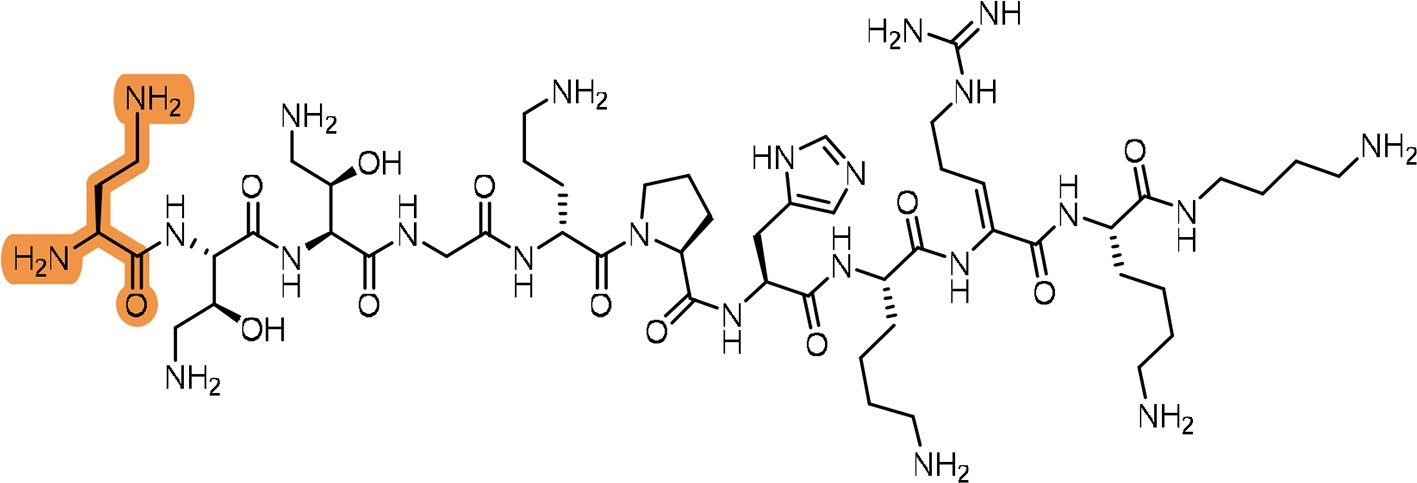	0.99	16	64 (× 4)
13	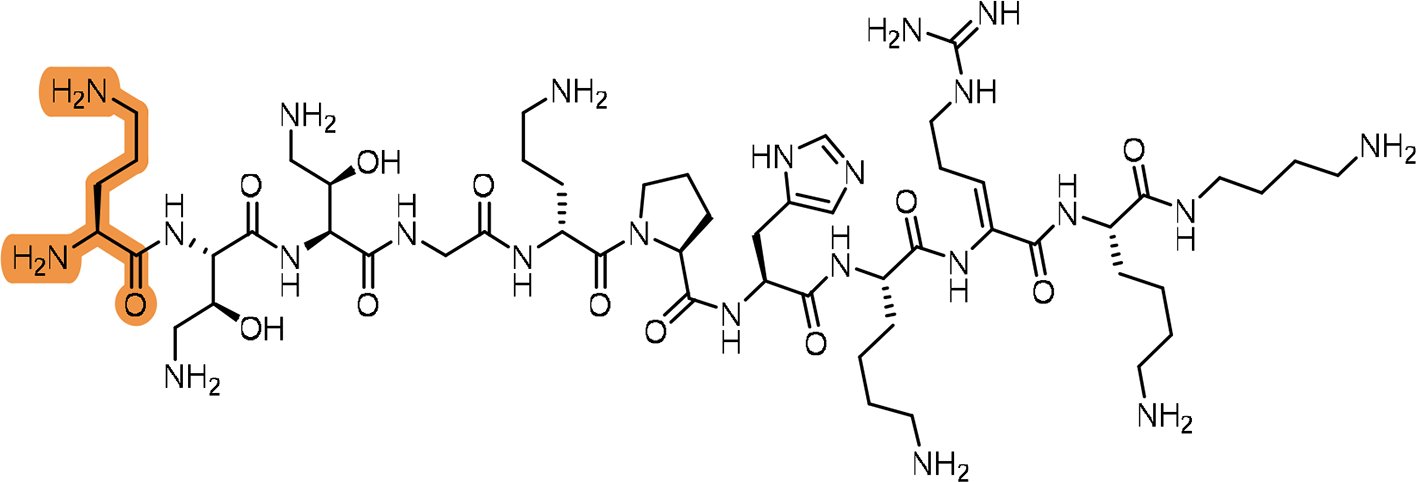	1.03	8	64 (× 8)
14	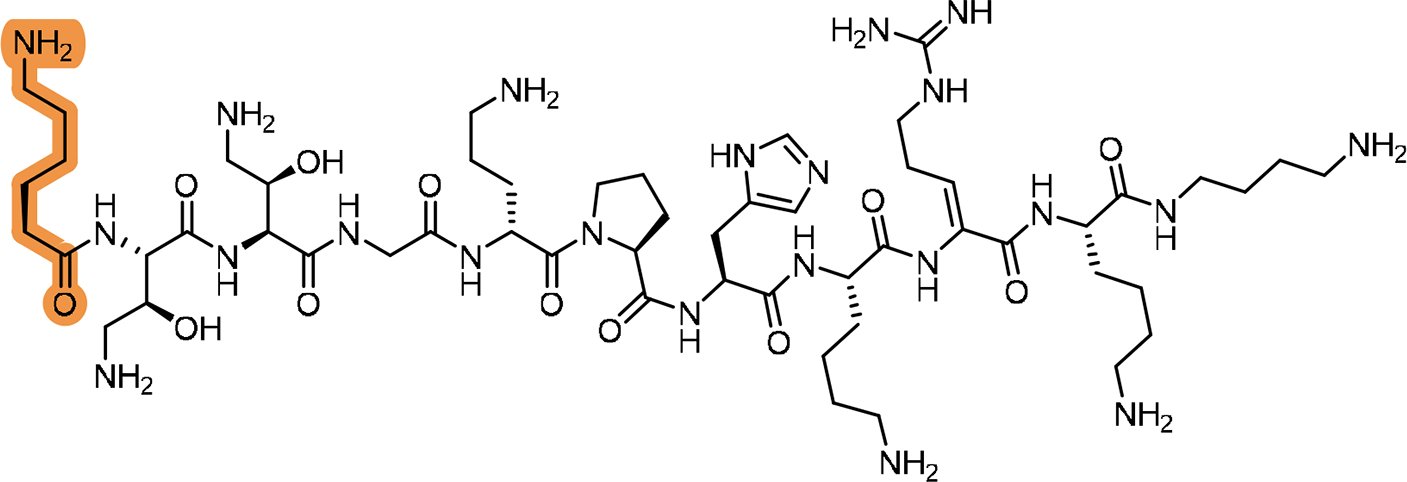	106.0	>512	>2,048 (nd)
15	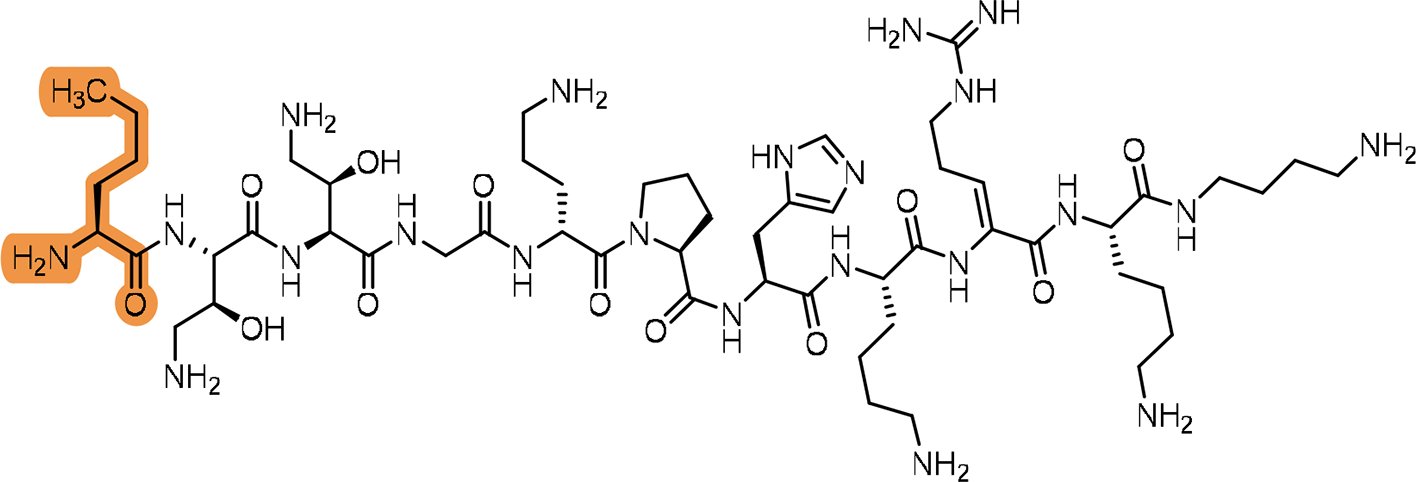	2.05	128	1,024 (× 8)
16	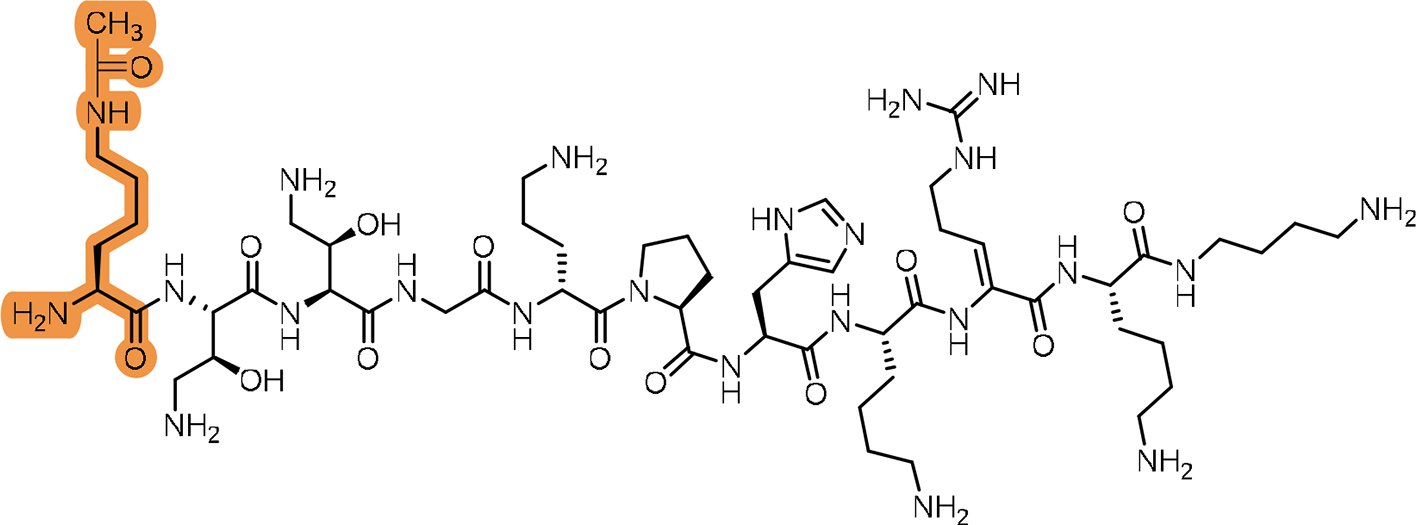	3.04	128	2,048 (× 16)

^*a*^ Data include IC_50_ values determined by an *E. coli in vitro* transcription-translation (IVTT) assays and minimum inhibitory concentrations (MICs) against *E. coli* XL1 (pUC19) (− OatA) and against *E. coli* XL1 (P*_lac_-oatA*-Xn) (+ OatA). Values in red parentheses represent the MIC ratio (fold-increase) between OatA-expressing and non-expressing *E. coli* XL1 strains. nd, not determined.

Removal of the *N*-terminal amine of Lys1 (analog **14**) resulted in a loss of both target inhibition and antibacterial activity against both strains, precluding any conclusion on its contribution to OatA-mediated inactivation. Removal (analog **15**) or acetylation (analog **16**) of the ε-amine reduced target potency and antibacterial activity, yet both analogs displayed diminished OatA-dependent MIC shifts of 8- and 16-fold, respectively, compared to 64-fold for NOSO-95C, indicating decreased susceptibility to OatA-mediated inactivation (Table 2).

Taken together, these N-terminal modifications show a graded effect: progressively shortening the Lys1 side chain (analogs 12 and 13) or neutralizing its terminal amine (analogs 15 and 16) reduced, but did not fully abolish, the OatA-dependent MIC shift, whereas complete removal of the side chain (analog 1) abolished it entirely. This indicates that both the length and the terminal amine functionality of the N-terminal residue contribute cumulatively to OatA-mediated inactivation, with the full lysine side chain being required for maximal susceptibility.

### OatA-mediated acetylation abolishes translation inhibition by NOSO-95C but not by analogue 1

**Fig 2:**
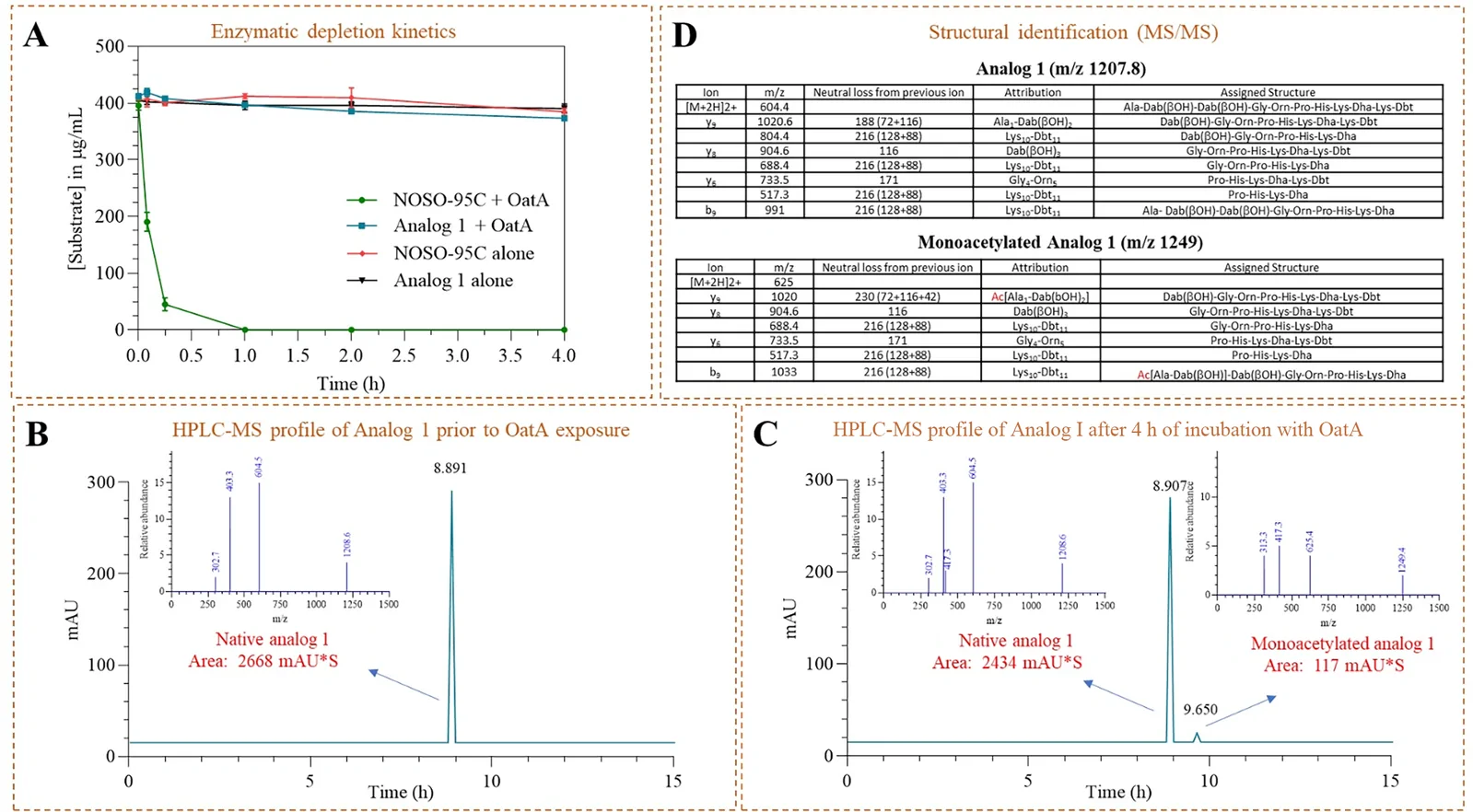
Enzymatic stability and structural characterization of analog 1 following OatA exposure. (**A**) Enzymatic depletion kinetics: Time-course monitoring of analog 1 and NOSO-95C (400 µg/mL) in the presence of purified OatA (30 µg/mL) and acetyl-CoA (750 µM). Samples were analyzed at 0, 5, 15, 30, 60, 120, and 240 min. Stability controls without the enzyme are represented by black upward-pointing triangles (NOSO-95C – OatA) and black downward-pointing triangles (analog 1 – OatA). Enzymatic reactions are represented by green circles (NOSO-95C + OatA) and blue squares (analog 1 + OatA). While NOSO-95C is completely depleted by OatA within 1 h, analog 1 remains mostly unacetylated after 4 h. Both compounds remained stable in the absence of the enzyme throughout the experiment. (**B**) HPLC-UV chromatogram (220 nm) and mass spectrum of analog 1 at *t* = 0: analysis prior to incubation showing the native peptide peak at m/z 1,207. (**C**) HPLC-UV chromatogram (220 nm) and mass spectrum of analog 1 after 4-h incubation with OatA: analysis showing the persistence of the native form (m/z 1,207) and the emergence of a minor monoacetylated peak (m/z 1,249). (**D**) MS/MS fragmentation patterns: comparison of MS/MS spectra for native analog 1 and its monoacetylated derivative, confirming the site of modification (acetylation = Ac in red).

To evaluate whether the absence of an OatA-dependent MIC shift for analog **1** resulted from impaired enzymatic acetylation, we evaluated the stability of NOSO-95C and analog **1** in the presence of purified OatA. First, calibration curves were established for both compounds using HPLC-UV detection at 220 nm. A linear correlation between concentration and peak area was demonstrated (Fig. S1). NOSO-95C and analog **1** (400 µg/mL) were then incubated with or without a determined concentration of purified OatA (30 µg/mL) in the presence of acetyl-CoA (750 µM) for various time lengths (0.08, 0.25, 0.5, 1, 2, and 4 h). The reaction progress was monitored by LC-MS analysis, measuring peak areas at 220 nm, and extrapolating concentrations from standard calibration curves. A rapid decrease of NOSO-95C concentration was measured, from 400 µg/mL to 0 µg/mL in 1 h, with a 50% decrease over 5 min (Fig. 2A). In contrast, analog **1** remained stable throughout the 4-h incubation period, maintaining over 95% of its initial concentration. (Fig. 2A).

Following 4-h incubation of analog 1 with OatA, two separate UV peaks were observed at retention times of 8.91 min and 9.65 min with associated m/z of 1,207 and 1,249, respectively, consistent with the masses of native and monoacetylated analog **1**. The peak area ratio at 220 nm (native/monoacetylated) was 20.8 (95.4% of native), indicating that the unacetylated form remained largely predominant (Fig. 2B and C). As previously described (17), the diacetylated form of NOSO-95C (m/z of 1,349) was predominant (92.8%), with a triacetylated form (m/z of 1,391) also present (7.2%). The native compound (m/z 1,265) had entirely disappeared (Fig. S2). The predominance of the diacetylated form of NOSO-95C over the triacetylated form, despite the presence of three acetylatable primary amines (Dab(βOH)2, Dab(βOH)3, Lys10), suggests that acetylation proceeds through a partially ordered, rather than fully stochastic, process. Two non-exclusive explanations can be proposed: (i) acetylation of the first two sites may sterically or electrostatically reduce the accessibility of the third amine within the OatA catalytic pocket; (ii) the 4-h incubation window, while sufficient to fully deplete the native compound, may not allow complete modification at the least favorable site. Distinguishing between these mechanisms would require monitoring the order of appearance of mono-, di-, and tri-acetylated species at early time points. Although this was beyond the scope of the present study, it represents a relevant avenue for future work on OatA catalytic mechanism.

To precisely map the site of enzymatic modification of analog 1, we conducted MS/MS fragmentation analysis on both the native and monoacetylated forms of analog **1** (Fig. 2D). Comparing the fragmentation patterns of the native peak (m/z 1,207) and the monoacetylated derivative (m/z 1,249) provided definitive evidence for the localization of the acetyl group. Notably, the y9 fragment ion, corresponding to the peptide chain from position 3 to 11, remained identical in both compounds (m/z 1,020). This identifies the Ala-Dab(βOH) dipeptide as the site of modification. Since the native dipeptide has a mass of 187 Da, the observed +42 Da shift in the monoacetylated precursor, combined with an identical m/z for the y9 ion, confirms the presence of an acetylated Ala-Dab(βOH) moiety (m/z 229).

**Fig 3:**
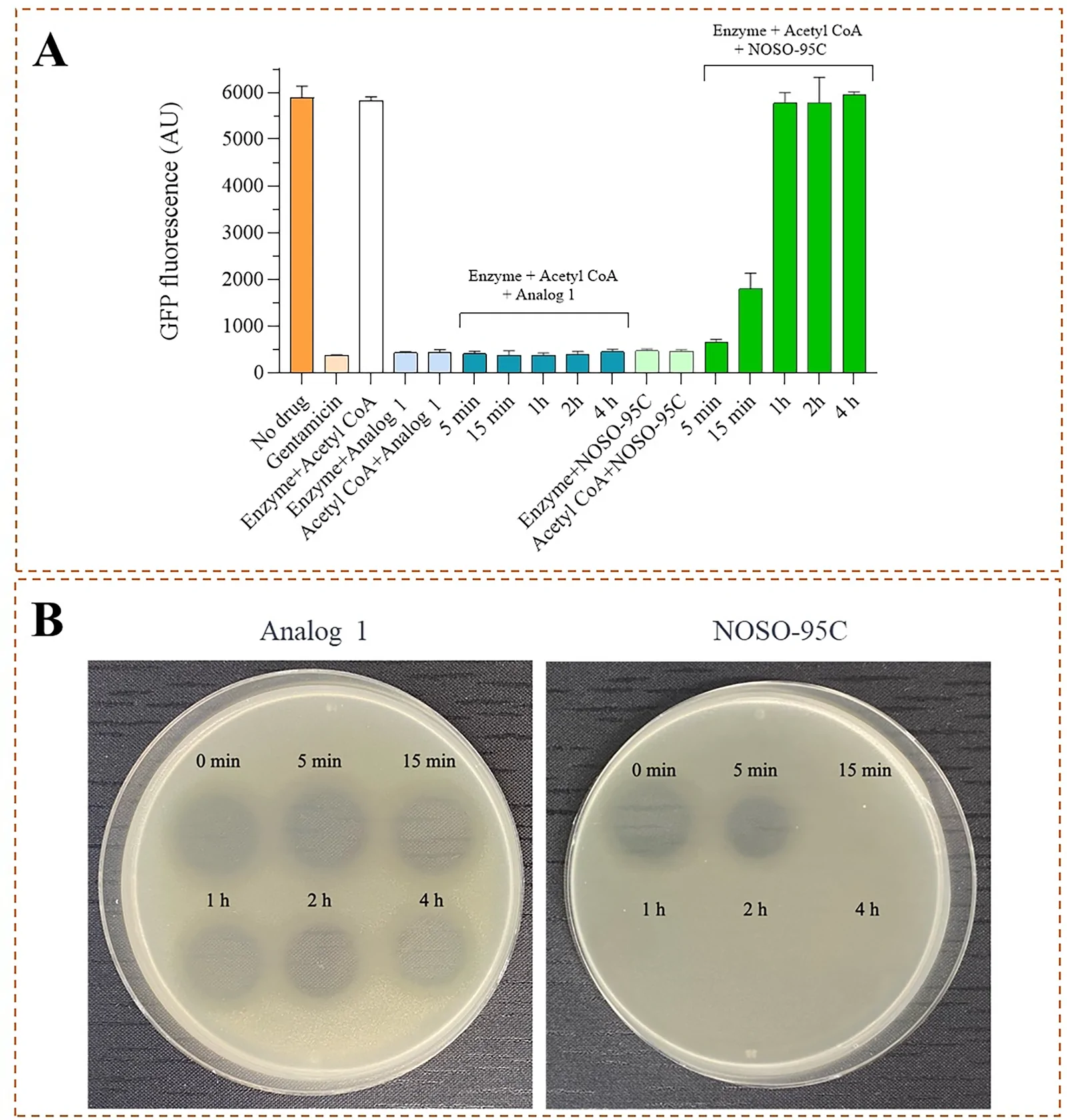
(**A**) *In vitro* translation inhibition. Assessment of GFP synthesis using an *E. coli* cell-free system following incubation of analog 1 and NOSO-95C (400 µg/mL) with OatA (30 µg/mL). Incubation times were 0.08, 0.25, 1, 2, and 4 h. Controls include: “no drug” corresponding to full reaction with no drug (maximum GFP production), gentamicin (10 µg/mL) served as a positive control, and reaction buffers containing enzyme and acetyl-CoA, or enzyme and analog 1. While the inhibitory activity of NOSO-95C decreases rapidly in a time-dependent manner, analog 1 maintains potent translation inhibition throughout the 4-h incubation. (**B**) Agar diffusion assay: Representative images of growth inhibition zones against *E. coli* ATCC 25,922. The spots correspond to 10 µL of reaction mixtures sampled at different time points (0 min to 4 h) during incubation of analog 1 and NOSO-95C with OatA (30 µg/mL) and acetyl-CoA (750 µM). Left plate: Analog 1 maintains stable antibacterial activity regardless of enzyme exposure time. Right plate: NOSO-95C shows a progressive reduction and complete loss of inhibition zones within 1 h, reflecting its rapid enzymatic inactivation.

The impact of OatA activity on analog **1** and NOSO-95C was evaluated using an *E. coli in vitro* coupled transcription–translation (IVTT) assay monitoring GFP production. We systematically analyzed samples from each incubation time point. In the absence of OatA, both compounds exhibited strong inhibitory activity, maintaining GFP fluorescence at a very low level (7.2% to 7.8% of the no-drug control, similar to gentamicin). In the presence of OatA, reaction mixtures with analog 1 maintained a strong inhibitory effect on GFP translation throughout the experiment (6.2% to 7.6% of the no-drug control), with no significant difference compared to gentamicin (Fig. 3A). This sustained activity is consistent with the near-absence of OatA-mediated acetylation of analog 1 documented above (Fig. 2A–C), confirming that the compound remains structurally intact and able to engage its ribosomal target even after prolonged enzyme exposure. On the other hand, we observed a rapid and time-dependent decrease in the inhibitory activity of NOSO-95C, with GFP fluorescence increasing to 30.5% at 15 min and reaching 98% of the control level after 1 h (Fig. 3A), mirroring its rapid enzymatic depletion (Fig. 2A).

These observations were validated using an agar diffusion antimicrobial assay with *E. coli* ATCC 25,922 (Fig. 3B). The antibacterial activity of supernatants of analog **1** in the presence of OatA remained remarkably constant, with clear inhibition zones maintaining a stable diameter throughout the 4-h experiment (≈16 mm). In contrast, supernatants of NOSO-95C in the presence of OatA exhibited a rapid and progressive loss of potency over time. After 5 min, the inhibition zone diameter narrowed from 16 mm to approximately 13 mm. By the 15-min mark, the clear zone was replaced by a faint and highly diffuse area of partial inhibition. This degradation led to a complete loss of any discernible antibacterial activity after 1 hour (Fig. 3B).

### Substrate specificity of OatA toward clinically used antibiotics

To assess whether OatA exhibits a narrow- or broad-spectrum activity, we evaluated its ability to inactivate a panel of structurally diverse primary amine-containing antibiotics, including aminoglycosides (gentamicin, amikacin, tobramycin), β-lactams (ampicillin, amoxicillin), and the polycationic lipopeptide polymyxin B. Antibacterial activity was assessed using spot-on-lawn assays against *E. coli* ATCC 25,922 following a 4-h incubation of each compound with purified OatA in the presence of acetyl-CoA. None of the antibacterials tested exhibited significant change in inhibition zone diameter after enzyme treatment compared to non-treated conditions, indicating that OatA does not measurably affect the activity of these antibiotics under the tested conditions (Table 3). We used NOSO-95C and analog **1** as controls. NOSO-95C exhibited a full loss of antibacterial activity (after incubation with OatA), with inhibition zones decreasing from 13.0 ± 0.0 mm to 0.0 ± 0.0 mm, while analog **1** retained full antibacterial activity after enzymatic treatment (12.3 ± 0.57 mm vs 12.0 ± 1.00 mm) (Table 3).

**TABLE 3:** Substrate specificity of OatA against clinically relevant amine-containing antibiotics^*a*^

Antibiotic	Number of primary amine groups	*E. coli* ATCC 25922			Comment
		MIC (µg/mL)	Zone diameter (mm), without OatA (mean ± SD, *n* = 3)	Zone diameter (mm), after 4-h incubation with OatA (mean ± SD, *n* = 3)	
Gentamicin	4-5	0.5	18.7 ± 0.57	18.7 ± 0.57	No effect of OatA
Amikacin	4	1	20.0 ± 0.0	19.7 ± 0.57	No effect of OatA
Tobramycin	5	1	17.0 ± 1.00	17.3 ± 0.0	No effect of OatA
Ampicillin	1	4	22.3 ± 0.57	22.0 ± 0.0	No effect of OatA
Amoxicillin	1	4	23.3 ± 0.57	22.7 ± 0.57	No effect of OatA
Polymyxin B	5	0.5	12.3 ± 0.57	11.7 ± 0.57	No effect of OatA
NOSO-95C	8	4	13.0 ± 0.0	0.0 ± 0.0	Complete loss of activity after OatA treatment
Analog **1**	7	8	12.3 ± 0.57	12.0 ± 1.00	No effect of OatA

^*a*^ Inhibition zone diameters (mm) were determined using spot assays (10 µL of mixtures containing 100 µg/mL of antibiotic solutions) on Mueller–Hinton agar seeded with *E. coli* ATCC 25922. For OatA treatment, antibiotics were incubated for 4 h with purified OatA (30 µg/mL) and acetyl-CoA (750 µM). Control samples (“without OatA”) were incubated under identical conditions in the presence of acetyl-CoA but without the enzyme. Values represent the mean ± standard deviation (*n* = 3). The number of primary amine groups potentially susceptible to acetylation is indicated for each compound.

It should be noted that, in contrast to the LC-MS/MS confirmation performed for NOSO-95C and analog 1, substrate turnover for this antibiotic panel was assessed functionally, at a single time point (4 h) and a single concentration (100 µg/mL), rather than by direct chemical detection of acetylated species. A low level of acetylation that does not translate into a measurable loss of antibacterial activity, as observed within the ODL series itself, cannot be formally excluded by this assay alone. This is acknowledged here as a limitation of the present study. The functional read-out nonetheless remains the most clinically relevant metric, and the absence of any detectable shift in inhibition zone across six chemically diverse, amine-rich antibiotics is consistent with genuine substrate exclusion rather than a marginal, assay-insensitive effect.

## DISCUSSION

In the present study, we used an alanine-scanning approach to investigate the structural determinants governing the susceptibility of NOSO-95C to inactivation by OatA, the producer-restricted self-resistance acetyltransferase of *X. nematophila* (17). We identified the *N*-terminal Lys_1_ residue as an essential determinant of this susceptibility: substitution of Lys_1_ with alanine abolished the OatA-mediated inactivation phenotype while preserving antibacterial activity and translation inhibition capacity. This conclusion is supported by three complementary lines of evidence. First, analog **1** exhibited identical MIC values against *E. coli* strains expressing or lacking OatA, in contrast to the 64-fold MIC shift observed for NOSO-95C. Second, LC-MS kinetic analyses demonstrated rapid depletion of NOSO-95C following incubation with purified OatA, whereas analog **1** remained largely unmodified after 4 h of exposure. Third, functional assays confirmed that analog **1** retained potent inhibition of bacterial translation and antibacterial activity after enzymatic treatment, while NOSO-95C rapidly lost activity.

The progressive reduction in OatA susceptibility observed with analogs **12**, **13**, **15**, and **16** further supports the importance of *N*-terminal side chain architecture. Shortening the side chain or neutralizing the ε-amine significantly reduced the resistance phenotype, although to a lesser extent than complete removal of the lysine side chain in analog **1**, indicating that both the length and charge distribution of the *N*-terminal residue contribute to efficient enzymatic processing. Interestingly, these modifications had only limited effects on translation inhibition activity, consistent with co-crystallization data showing that the N-terminal α-NH2 group, rather than the Lys1 side chain, forms the critical hydrogen bonds with the A-site tRNA (11). Our results show that while this α-NH_2_ is crucial for translation inhibition, the lysine side chain and its ε-NH₂ group are specifically required for OatA activity. By identifying this structural divergence, we effectively decoupled the requirements for potent translation inhibition from those governing enzymatic resistance. This dissociation offers an important opportunity for designing ODL analogs that retain ribosomal affinity while evading OatA.

While Lys_1_ itself is not the primary site of acetylation (17), this residue appears to play an indirect but essential role in substrate recognition by the enzyme or in productive enzyme-substrate interaction. One possible explanation is that the positively charged and conformationally flexible Lys_1_ side chain contributes to substrate positioning within the OatA catalytic pocket, thereby facilitating acetylation of more distal amino groups. Similar long-range recognition mechanisms have been described for other members of the GNAT superfamily, including AACs, in which substrate recognition depends not only on the presence of acetylatable amines but also on the global spatial organization and electrostatic properties of the substrate (19).

We also demonstrated the narrow substrate specificity of OatA. Despite the presence of multiple primary amines in clinically relevant antibiotics such as aminoglycosides (gentamicin, amikacin, tobramycin), β-lactams (ampicillin, amoxicillin), and polymyxin B, no significant reduction in antibacterial activity was observed following incubation with purified OatA under our experimental conditions. These results indicate that OatA does not behave as a broad-spectrum amine acetyltransferase but rather displays strong selectivity toward the ODL scaffold, consistent with its role as a dedicated self-resistance enzyme rather than a broadly disseminated resistance determinant. Such narrow substrate specificity contrasts with AACs, which have demonstrated remarkable evolutionary plasticity (20). An illustrative example is the AAC(6')-Ib-cr variant, which, through only two point mutations, expanded its activity beyond aminoglycosides to include fully synthetic fluoroquinolones such as ciprofloxacin (21).

These observations are consistent with the restricted genomic distribution of the *oatA* locus, which is confined to a limited group of entomopathogenic symbiotic bacteria (17). GNAT-family acetyltransferases are broadly recruited by antibiotic-producing bacteria as self-resistance determinants, detoxifying their own specialized metabolites, as exemplified by EdeQ-mediated resistance to edeine (5) and the recently described self-resistance acetyltransferase conferring producer immunity to the lasso peptide lariocidin (22). OatA appears to have evolved as a specialized self-protection mechanism. This mechanism is tightly coupled to ODL biosynthesis, rather than serving as a determinant with potential for broader clinical dissemination.

### Conclusion

We identified Lys1 at the *N*-terminal part of NOSO-95C as the primary molecular determinant governing susceptibility of ODLs to inactivation by OatA, a narrow-spectrum, producer-restricted self-resistance acetyltransferase. While OatA acetylates multiple positions on NOSO-95C, modifying or removing Lys1 side chain, as seen in analog **1**, markedly reduces susceptibility to enzymatic inactivation without compromising ribosomal target engagement. Moreover, OatA shows no measurable activity against other amine-containing clinical antibiotics such as aminoglycosides, β-lactams, or polymyxins, reinforcing its status as a dedicated self-resistance enzyme rather than a broad-spectrum resistance threat. These findings provide a clear structure-activity strategy for the rational design of next-generation ODLs that evade this specific enzymatic inactivation mechanism while maintaining potent ribosomal inhibition.

## MATERIALS AND METHODS

### Bacterial strains, plasmids, and growth conditions

The strain and plasmids, *E. coli* XL1(pUC19) and *E. coli* XL1 (P*_lac_-oatA*-Xn), used in this study are described in reference 17. *E. coli* ATCC 25922 was purchased from the American Type Culture Collection (ATCC). Bacteria were routinely grown in Luria-Bertani (LB) medium at 37°C.

### Antimicrobial agents

NOSO-95C and analogs were synthesized at Nosopharm (Nîmes, France) as described in reference 12. Gentamicin (G3632, Sigma-Aldrich), amikacin (A1774, Sigma-Aldrich), tobramycin (T4014, Sigma-Aldrich), ampicillin (A9518, Sigma-Aldrich), amoxicillin (A8523, Sigma-Aldrich), and polymyxin B (P0972, Sigma-Aldrich) were provided as standard powders by the manufacturers.

### Minimum inhibitory concentration (MIC)

MICs were determined in triplicate, in accordance with CLSI guidelines but with modifications for *E. coli* constructs (23). A modified method of inoculum preparation was used in which about 10 colonies were picked from MH agar supplemented with 100 μg/mL of ampicillin and used to inoculate 10 mL of MHB II supplemented with 100 μg/mL of ampicillin. The inoculum was incubated for 1.5 to 2 h at 35°C, with shaking at 150 rpm, until the OD600 reached 0.15 to 0.25. IPTG was added at a concentration of 0.2 mM for induction, and the culture was incubated for 30 min at 35°C, with shaking at 150 rpm. The inoculum was then diluted 1:20 in saline to obtain the final inoculum. The reaction medium consisted of MHB II supplemented with 0.2 mM IPTG.

### *E. coli* cell-free transcription-translation assay

The effect of test compounds on *in vitro* bacterial protein synthesis was tested in the Expressway Cell-Free *E. coli* Expression System (Invitrogen). The *gfp* gene was amplified from the pCmGFP plasmid (24), cloned into pEXP5-CT TOPO vector (Thermo Fisher), and the resulting plasmid was used as a template for *in vitro* transcription-translation. The reactions were assembled following the manufacturer’s protocol and carried out in 50 µL in the wells of a polystyrene black 96-half-well microplate (Greiner), including the addition of feed buffer at 30 min of incubation to support optimal protein synthesis. Reactions were initiated by adding 1 µg of plasmid DNA, plates were incubated at 30°C, and fluorescence was measured every 20 min (λ_ex_ = 475 nm, λ_em_ = 520 nm) with a microplate reader. IC_50_ values were calculated at 1 h after the addition of feed buffer using GraphPad Prism 6 Software (GraphPad Software, San Diego, CA, USA). In a second set of experiments, fluorescence from GFP synthesis was recorded at 20 min after the addition of feed buffer. Gentamicin was used as a positive control at 10 μg/mL.

### Cloning, overexpression, and purification of OatA

The *oatA* gene was cloned, overexpressed in *E. coli* BL21(DE3), and the His-tagged protein purified by Ni-NTA affinity chromatography as previously described in (17).

### *In vitro* acetylation of analogue 1, NOSO-95C, and antibiotics

*N*-Acetylation of compounds was performed in a polystyrene microtube with purified His-tagged OatA diluted to 30 μg/mL in 75 mM HEPES (pH 7.5) supplemented with glycerol (5.7% [vol/vol]), 400 µg/mL analog **1** or NOSO-95C, and 750 μM acetyl-CoA in 75 mM HEPES (pH 7.5) for 4 h at 28°C. Aliquots were sampled at 0.08, 0.25, 1, 2, and 4 h.

In a second set of experiments, the same protocol was performed using NOSO-95C, analog **1**, and a panel of six antibiotics at a final concentration of 100 μg/mL. After 4 h of incubation, samples were directly used for antibacterial activity assays on agar plates.

### LC-MS analysis of analogue 1, NOSO-95C, and acetylated derivatives

NOSO-95C, analog **1**, and the acetylated derivatives were analyzed by LC-MS. Analyses were performed on an Agilent 1260 Infinity HPLC system. The HPLC column used was a Waters Symmetry analytical C18 column (5 µm, 4.6 mm×150 mm) maintained at 25°C. Solvent A was H_2_O plus 0.1% (vol/vol) trifluoroacetic acid, solvent B was acetonitrile, and the flow rate was 0.7 mL/min. The mobile phase composition was 98% solvent A ramped to 30% solvent B at 15 min. Sample injection volume was 10 μL. UV detection was performed at 220, 230, and 250 nm.

Quantification was performed using external calibration curves generated with analog **1** and NOSO-95C at concentrations ranging from 25 to 2000 μg/mL. Peak areas were extracted at 220 nm. Calibration curves were linear over this concentration range.

ESI-LC-MS data were obtained in positive mode on an Agilent 1260 Infinity system (Agilent 6120 Quadrupole LC/MS, 1260 Quaternary Pump, 1260 ALS, 1260 TCC, 1260 DAD VL, 1260 FC-AS). An ion spray (IS) voltage of 3,000 V, a source temperature of 140°C, a desolvation temperature of 450°C, a declustering potential (DP) of 30 V, and a collision energy (CE) in the range of 20 to 60 V were applied.

### Antibiotic activity

The antibacterial activity of the *in vitro* acetylated samples was assessed using an agar overlays assay. Briefly, an overnight culture of *E. coli* ATCC 25,922 grown in LB broth at 35°C was mixed with molten Mueller–Hinton II supplemented with 1.4% agarose at 2% (vol/vol) and poured into Petri dishes. After solidification, 10 μL of each sample was spotted onto the surface of the agar. Plates were allowed to dry and incubated at 35°C for 20 h. Zones of growth inhibition were then recorded.

## SUPPLEMENTAL MATERIAL

Supplemental figures aac.00743-26-s0001.pdf Fig. S1 and S2.

ASM does not own the copyrights to Supplemental Material that may be linked to, or accessed through, an article. The authors have granted ASM a non-exclusive, world-wide license to publish the Supplemental Material files. Please contact the corresponding author directly for reuse.
