# Supplementary material for: N-terminal lysine as a critical determinant of odilorhabdin susceptibility to inactivation by the self-resistance acetyltransferase OatA
Source: Antimicrob Agents Chemother. 2026 Sep 16;70(10):e00743-26. doi: 10.1128/aac.00743-26 (PMC13689418; doi:10.1128/aac.00743-26)
Supplement: Supplementary file 1 [file aac.00743-26-s0001.pdf]

Supplemental Material

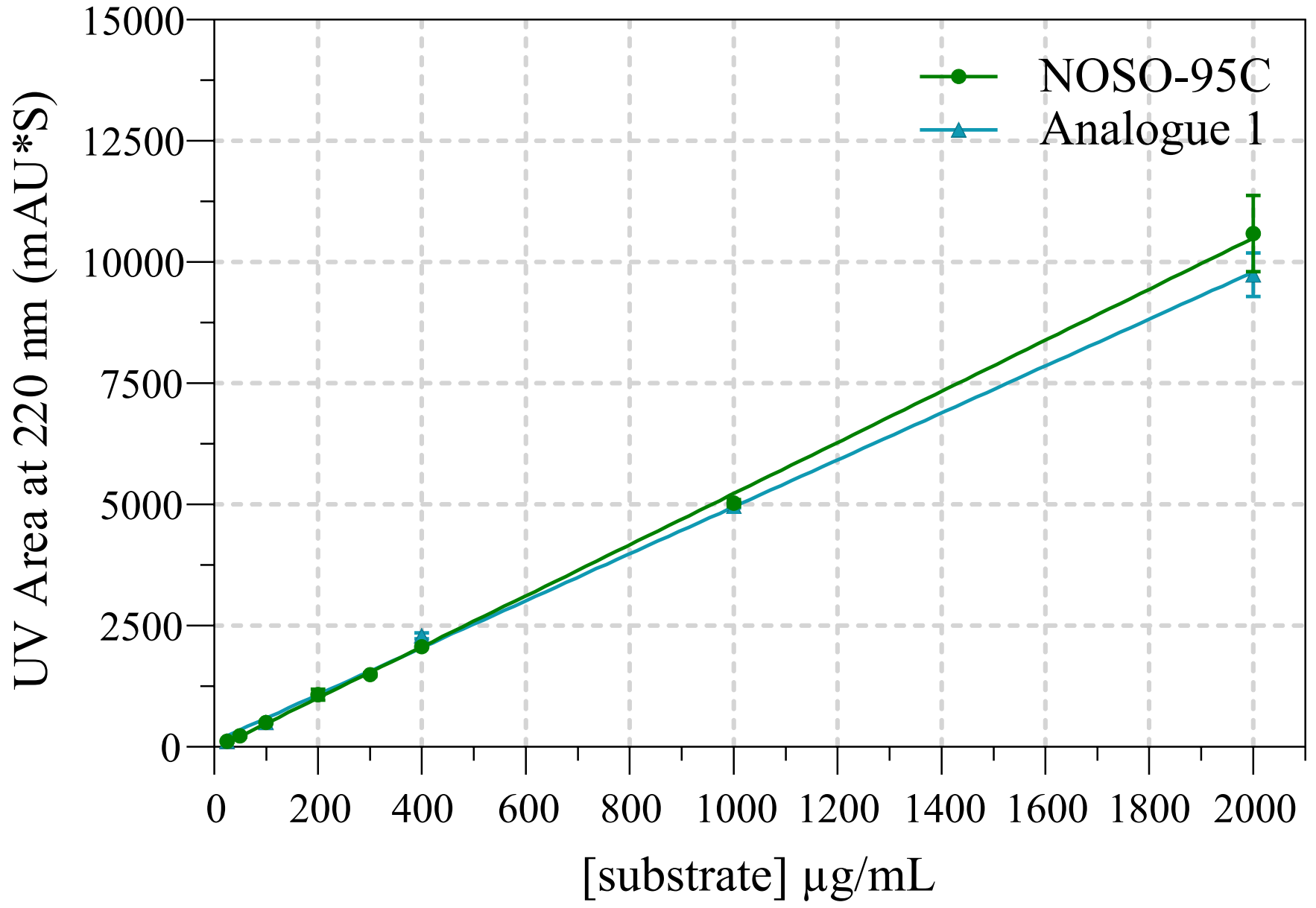

Supplemental data Figure S1: Standard Calibration Curves: HPLC-UV chromatograms at 220 nm showing the linear correlation between concentration and peak area for analogue **1** and NOSO-95C (n =3).

Retention time (min)	Peak area (220 nm)	Ion mass	Suggested compound
9.67	1713	[M+H]+:1349.7<br>[M+2H]2+: 675.0<br>[M+3H]3+: 450.3<br>[M+4H]4+: 338.0	Diacetylated NOSO-95C
10.41	132	[M+H]+:1391.2<br>[M+2H]2+: 695.8<br>[M+3H]3+: 464.3<br>[M+4H]4+: 348.4	Triacetylated NOSO-95C

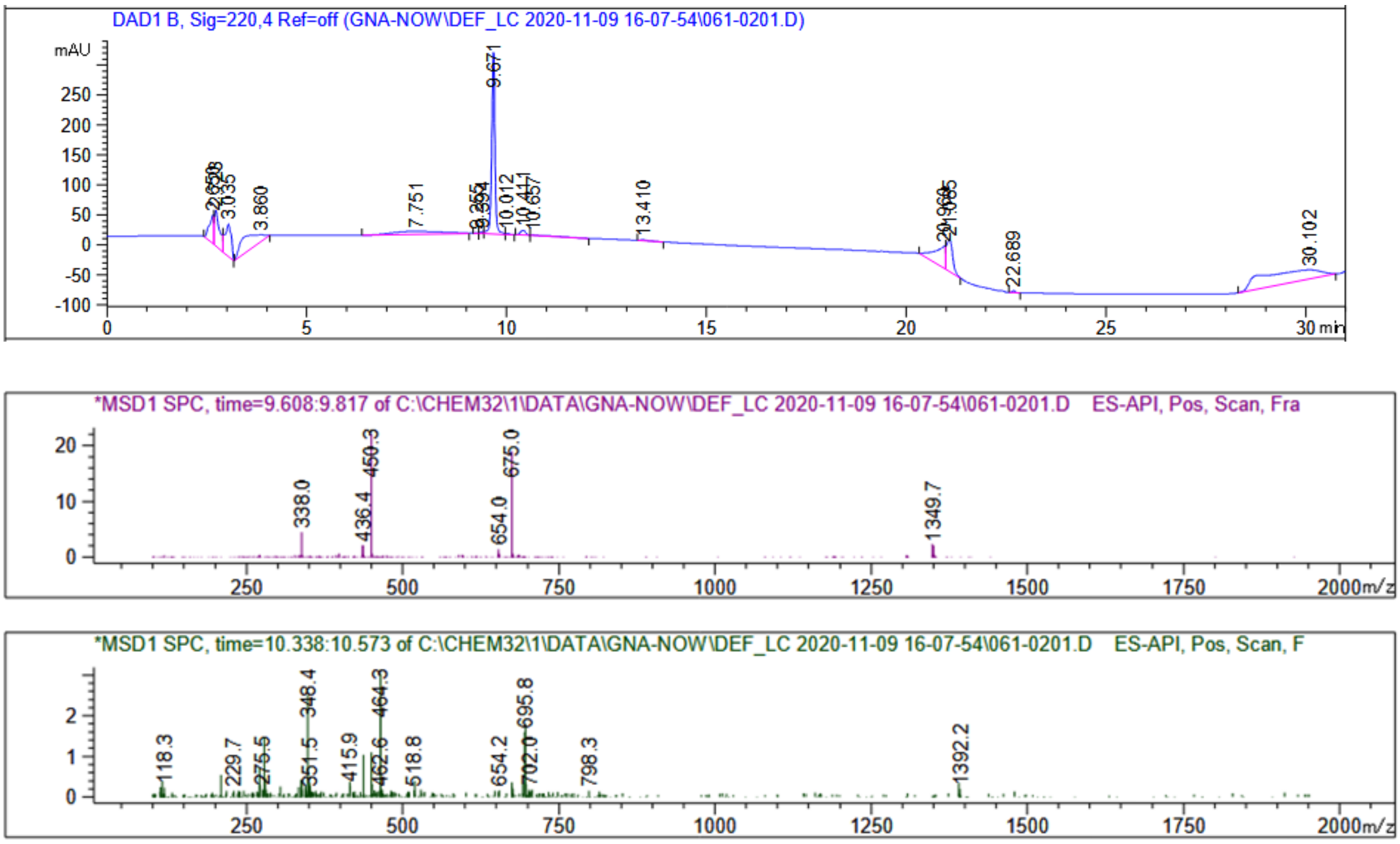

Supplemental data Figure S2. LC-MS analysis of NOSO-95C and acetylated derivatives after a 4h *in vitro* acetylation (see Materials and Methods) with Acetyl CoA, and Odilorhabdin acetyltransferase A (OatA). A table and corresponding LC-MS chromatograms present observed ions with retention time, peak area and suggested compounds.
